# The JIP1 Scaffold Protein Regulates Axonal Development in Cortical Neurons

**DOI:** 10.1016/j.cub.2008.01.025

**Published:** 2008-02-12

**Authors:** Federico Dajas-Bailador, Emma V. Jones, Alan J. Whitmarsh

**Affiliations:** 1 Faculty of Life Sciences, Michael Smith Building, University of Manchester, Oxford Road, Manchester, M13 9PT, United Kingdom

**Keywords:** SIGNALING

## Abstract

The development of neuronal polarity is essential for the determination of neuron connectivity and for correct brain function. The c-Jun N-terminal kinase (JNK)-interacting protein-1 (JIP1) is highly expressed in neurons and has previously been characterized as a regulator of JNK signaling. JIP1 has been shown to localize to neurites in various neuronal models, but the functional significance of this localization is not fully understood [1–4]. JIP1 is also a cargo of the motor protein kinesin-1, which is important for axonal transport [2, 4]. Here we demonstrate that before primary cortical neurons become polarized, JIP1 specifically localizes to a single neurite and that after axonal specification, it accumulates in the emerging axon. JIP1 is necessary for normal axonal development and promotes axonal growth dependent upon its binding to kinesin-1 and via a newly described interaction with the c-Abl tyrosine kinase. JIP1 associates with and is phosphorylated by c-Abl, and the mutation of the c-Abl phosphorylation site on JIP1 abrogates its ability to promote axonal growth. JIP1 is therefore an important regulator of axonal development and is a key target of c-Abl-dependent pathways that control axonal growth.

## Results and Discussion

### JIP1 is Localized at Axonal Growth Cones

**Figure 1:**
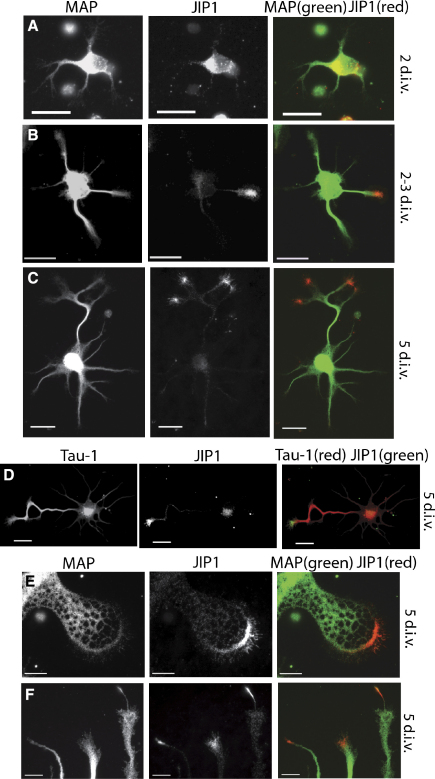
JIP1 Is Enriched in Nascent and Mature Axonal Growth Cones Cortical neurons were fixed at 2 and 5 d.i.v. and stained with anti-MAP, anti-JIP1, or anti-Tau-1. (A) At 2 d.i.v., the majority of neurons have JIP1 (red) localized in the cell body, with a diffuse cytoplasmic distribution. (B) By the end of stage 2 of neuronal development, JIP1 (red) localized to a single neurite before any of the processes were morphologically distinct. (C) In maturing axons, JIP1 (red) localized to branching axonal processes but was absent from dendrites. Scale bars represent 20 μm. (D) After polarization, the axonal localization of JIP1 was confirmed by immunostaining for Tau-1. JIP1 (green) only localized to Tau-1 (red)-positive axons. Scale bars represent 20 μm. (E and F) Growth cones stained with anti-JIP1 (red) and anti-MAP (green). In lamelipodial growth cones, JIP1 localized to a limited area of the transition and peripheral regions. This was also observed in filopodial axonal structures, where JIP1 staining was detected at the protrusion of turning filopodiums. Scale bars represent 10 μm.

To identify the potential role of JIP1 in neurite development, we monitored its localization during the polarization of cortical neurons in culture. It has previously been established that from 1 to 5 days in vitro (d.i.v.), neurons progress from a nonpolarized state toward a structure with a single differentiated axon and several shorter dendrites [5]. In the present study, at 2–3 d.i.v., the majority of neurons had developed several short neurites of similar length and morphology (Figures 1A and 1B). Initially, JIP1 localized to the cell body of these nonpolarized neurons (Figure 1A), but it subsequently relocalized to the tip of a single neurite (Figure 1B). At 5 d.i.v., the neurons were asymmetric in shape, with a single long axon and several shorter dendrites. At this and later developmental stages, JIP1 was specifically localized to the tip of the axon and its newly developed branches (Figure 1C). The axonal localization of JIP1 was confirmed by colabeling with the axonal marker Tau-1 [6] (Figure 1D). Interestingly, in nonpolarized neurons, the JIP1 localization to a single neurite precedes both morphological differentiation of the axon and segregation of Tau-1 (Figure 1, and Figure S1 available online), indicating that JIP1 is a very early marker for axons. The timing and specificity of JIP1 localization is coincident with that of kinesin-1 [7] and suggests that the association between kinesin-1 and JIP1 could be one of the early steps that determines the molecular identity of nascent axons. This is supported by a recent study that demonstrates that the binding of JIP1 and fasciculation and elongation protein ζ1 (FEZ1) to kinesin-1 is sufficient to activate the motor protein for microtubule binding and motility [8].

Within the axonal growth cone, JIP1 specifically localizes to the transition and peripheral regions and appears to track the turn of the growth cone (Figures 1E and 1F). Moreover, the disruption of cytoskeleton structures indicates that JIP1 is preferentially associated with dynamic microtubules capable of protruding into peripheral growth-cone areas (Figure S2).

### JIP1 Is Required for Axonal Development

**Figure 2:**
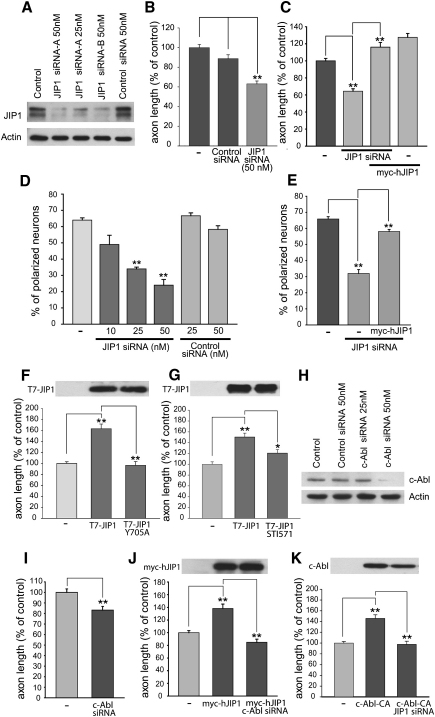
JIP1 Regulates Axon Length and Neuronal Polarization (A) Immunoblot demonstrating a reduction in JIP1 protein levels with the use of two different JIP1 siRNAs but not with nonspecific control siRNA (72 hr after transfection). (B) Axonal length of cortical neurons was evaluated 72 hr after cotransfection with JIP1 siRNA and eGFP. (C) The decrease in axonal length after JIP1 siRNA was rescued by expression of myc-tagged human JIP1. (D) Measurement of the number of polarized neurons in developmentally delayed cortical neurons (see Supplemental Data) transfected with JIP1 siRNA and eGFP. (E) Ectopic expression of myc-tagged human JIP1 rescues the decrease in neuronal polarization observed with JIP1 siRNA. For polarization experiments, data are expressed as percent of total number of neurons (mean ± standard error of the mean [SEM], from six independent experiments and ∼250 neurons counted for each condition). (F, G, and I–K) Neurons were transfected with constructs expressing either wild-type JIP1, a JIP1 mutant that does not bind to kinesin-1 (JIP1-Y705A), c-Abl siRNA, myc-hJIP1, constitutively active c-Abl (c-Abl-CA), or JIP1 siRNA as indicated. Where indicated, the c-Abl inhibitor STI-571 (1 uM) was added to cultures 14 hr prior to cell lysis. JIP1 expression levels were detected with anti-T7 and anti-myc tag antibodies, and c-Abl was detected with a c-Abl antibody. (H) Immunoblot for c-Abl protein levels after transfection with c-Abl siRNA. For all measurements of axonal length, the data are expressed as percent of respective controls (mean ± SEM, from six to eight independent experiments and ∼100 axons measured for each condition). Statistical significance was determined via one-way ANOVA, with post hoc Tukey's test for comparison between groups. Values of p < 0.05 (^∗^) were taken to be statistically significant, (^∗∗^) indicates values of p < 0.001.

Given its localization, it was important to determine whether JIP1 was required for axonal development. Cortical neurons were independently transfected with two different JIP1 siRNA duplexes (siRNA-A and siRNA-B) prior to plating, and a significant knockdown of JIP1 protein levels was detected by both immunofluorescent staining (Figure S4A) and immunoblotting (Figure 2A). The JIP1 siRNA-A caused a marked reduction in axonal length compared to the control siRNA (Figure 2B). Similar results were obtained with JIP1 siRNA-B (data not shown). Importantly, the decrease in axonal length observed after the knockdown of JIP1 was rescued with a construct expressing human JIP1, which is not targeted by the mouse-specific siRNA (Figure 2C). We also observed that the knockdown of JIP1 caused a reduction in the number of axonal branches (Figure S3), although this could be interpreted as an indirect consequence of the decrease in overall axonal length. Unlike the effect observed on axons, the length of dendrites was not modified by the JIP1 siRNA (control, 48.0 μm ± 0.85 versus JIP1 siRNA, 47.7 μm ± 0.82).

The requirement of up to 48 hr for the siRNA to have a significant effect on protein levels made it difficult to analyze the role of JIP1 in axonal specification. To circumvent this problem, we transfected neurons with JIP1 siRNA and plated them on uncoated dishes, thereby preventing axonal development. After 24 hr, cells were collected and cultured as normal on poly-L-ornithine-coated dishes. In these conditions, the JIP1 siRNA led to a concentration-dependent decrease in neurons displaying a distinguishable axon (Figure 2D), and this was reversed by the ectopic expression of human JIP1 (Figure 2E). Thus, JIP1 is necessary for neuron polarization and axonal growth. It is currently not clear how the processes of axonal specification and growth relate in vivo. Emerging evidence suggests that growth might not be just a consequence of axonal specification [9]. For example, the ablation of axons to eliminate length differences can reset axon-dendrite polarity [10], and faster neurite growth can specify an axon [11], indicating that promotion of axonal growth could contribute intrinsically to axonal specification.

The ectopic expression of JIP1 leads to an increase in axonal length, confirming an important role for JIP1 in axonal growth (Figures 2F, 2G, and 2J and Figure S4B). This was dependent on kinesin-1-mediated axonal transport of JIP1 because neurons transfected with a construct expressing a JIP1 mutant that is unable to bind to kinesin-1 (JIP1-Y705A, [2]) did not display increased axonal growth (Figure 2F and Figure S4C). Considering the established role of JIP1 as a JNK scaffold protein [1], we also expressed a JIP1 mutant that was unable to bind to JNK (JIP1-R160G/P161G, [12]). This mutant promoted axonal elongation similar to wild-type JIP1 (Figure S5), indicating that JNK binding to JIP1 is not essential for axonal growth. However, we cannot rule out the possibility that JIP1 may regulate some aspects of JNK signaling during axonal growth because of its ability to bind other components of JNK modules.

Previous studies to address the function of JIP1 with knockout mice have produced different phenotypes [13], with one study resulting in preimplantation lethality [14] and others revealing a stress-related neuronal phenotype with defects in JNK signaling and neuronal apoptosis [1, 15]. It is possible that in some mouse-strain backgrounds in vivo, other members of the JIP family might help restore neuronal functionality in the JIP1 knockout mice. Indeed, transgenic expression of JIP1 partially rescued axon-guidance defects of JIP3-deficient brains, demonstrating the likely existence of compensatory mechanisms [16].

### c-Abl Phosphorylation of JIP1 Regulates Axonal Growth

Given that axonal development is a complex process involving a variety of signaling processes [17], we anticipated that some of these may regulate JIP1 function. Deploying pharmacological inhibitors of signaling pathways, we identified the c-Abl tyrosine kinase as a candidate regulator of JIP1 function in axonal growth (Figure 2G and data not shown). Previously, c-Abl has been proposed to link neuronal receptors and the cytoskeletal regulatory machinery [18–20] and to promote dendrogenesis, neurite growth, and the emergence of branching microspikes [19, 21, 22]. Our results, from use of the c-Abl protein kinase inhibitor STI571 and c-Abl siRNA, demonstrate that the promotion of axonal elongation by JIP1 was dependent on c-Abl (Figures 2G–2J). In addition, the expression of a constitutively active form of c-Abl (c-Abl-CA) led to an increase in axonal length that was significantly diminished by JIP1 siRNA (Figure 2K). These data indicate that JIP1 is likely to be a downstream component of a c-Abl-dependent signaling pathway controlling axonal growth.

**Figure 3:**
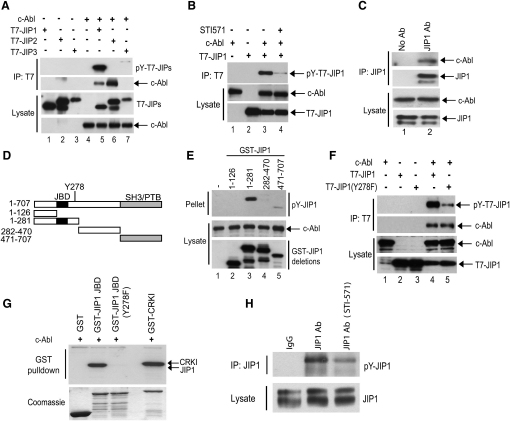
c-Abl Binds to JIP1 and Phosphorylates It at Tyr278 (A and B) Constructs expressing c-Abl and either JIP1, JIP2, or JIP3 were introduced into COS-7 cells. JIP-containing complexes were immunoprecipitated with the anti-T7 tag antibody and c-Abl was detected with a c-Abl antibody. JIP phosphorylation was detected with phosphotyrosine antibodies. The c-Abl inhibitor STI-571 (10 μM) was added to cultures 14 hr prior to cell lysis. (C) Endogenous JIP1 was immunoprecipitated from NIE-115 cells and the presence of c-Abl in immunocomplexes detected using a c-Abl antibody. (D) Schematic representation of the JIP1 constructs used for mapping the c-Abl phosphorylation site (JBD: JNK binding domain; SH3: SRC homology domain-3; PTB: phosphotyrosine binding domain). (E) GST-tagged JIP1 constructs were introduced into COS-7 cells along with c-Abl-CA and GST-containing complexes were isolated from cell lysates with glutathione-sepharose beads and the presence of Tyr-phosphorylated JIP1 analyzed. (F) Constructs expressing T7-JIP1 and T7-JIP1-Y278F were introduced into COS-7 cells with or without a c-Abl expressing vector. JIP1 containing complexes were immunoprecipitated using anti-T7 tag antibody and the presence of c-Abl and Tyr-phosphorylated JIP1 were analyzed. (G) In vitro phosphorylation of JIP1 by c-Abl. GST-tagged JIP1 JBD (aa 127-281) and the Y278F mutant were incubated with recombinant c-Abl in protein kinase assays. GST-CRKI was used as a positive control. Autoradiograph (top panel) and coomassie stained SDS-PAGE gel (lower panel) are shown. (H) Endogenous JIP1 was immunoprecipitated from cortical neurons incubated with or without STI571 (1 μM, 6 hr), and Tyr phosphorylation was detected with phosphotyrosine-specific antibodies.

To uncover the mechanism underlying the observed link between c-Abl signaling and JIP1 function, we performed coimmunoprecipitation studies from transfected COS-7 cells. These revealed that JIP1 associates with c-Abl and that c-Abl promotes JIP1 tyrosine phosphorylation (Figures 3A and 3B). There was no significant tyrosine phosphorylation of the JIP family members JIP2 and JIP3 (Figure 3A). To identify the JIP1 residues phosphorylated by c-Abl, we expressed GST-tagged fragments of JIP1 along with c-Abl (Figures 3D and 3E). A JIP1 fragment comprising residues 1–281 showed significant tyrosine phosphorylation (Figure 3E), and sequence analysis (Scansite 2.0, [23]) indicated that Tyr278 was a candidate phosphorylation site. We subsequently generated a JIP1 construct with a Tyr to Phe mutation at residue 278 (JIP1-Y278F), and although this mutant still bound to c-Abl, the tyrosine phosphorylation was significantly reduced (Figures 3F and 3G). Importantly, we could detect endogenous complexes of JIP1 and c-Abl in neuronal cells (Figure 3C) and demonstrate that endogenous JIP1 was phosphorylated on Tyr in a c-Abl-dependent manner in cortical neurons (Figure 3H).

Although our data strongly point to a key involvement of c-Abl in the regulation of JIP1 function in axonal development, it was possible that the homologous c-Abl-related-gene kinase (Arg) could play a role. Indeed, Arg is capable of phosphorylating JIP1 at Tyr278 (Figure S6A), although the specific knockdown of Arg protein levels did not appear to affect basal axonal growth and only caused a minor decrease in the growth induced by the ectopic expression of JIP1 (Figures S6B and S6C). A recent study has demonstrated that Src family kinases can also phosphorylate JIP1 on tyrosine and regulate JNK-module activation [24]. However, the functionally important sites described in that study were distinct from the c-Abl site identified here, and the inhibition of Src kinases did not prevent the increase in axonal length mediated by JIP1 (data not shown). Therefore, our data suggest that c-Abl, rather than Arg or Src, is likely to play a predominant role in regulation of JIP1 during axonal development.

**Figure 4:**
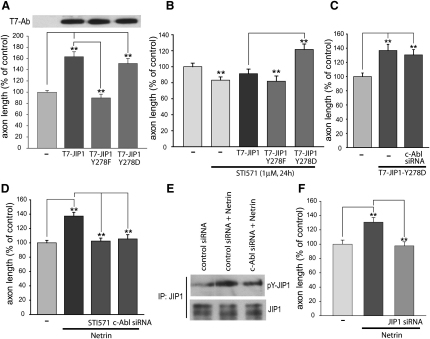
JIP1-Dependent Axonal Growth Requires Tyr278 Phosphorylation (A and B) Cortical neurons were cotransfected with an eGFP-expression vector plus the indicated JIP1-expression vectors, and the axonal length of neurons was evaluated 72 hr later. Neurons were incubated with STI571 for 24 hr prior to fixation. (C) Axonal length of cortical neurons after expression of JIP1-Y278D plus or minus c-Abl siRNA. (D) Axonal length was measured after incubation of neurons with netrin-1 (150 ng/ml, 12 hr), plus or minus STI571 (1 μM) or c-Abl siRNA (50 nM). (E) Tyr phosphorylation of endogenous JIP1 immunoprecipitated from cortical neurons transfected with control siRNA or c-Abl siRNA (50 nM) for 72 hr and stimulated with netrin-1 (150 ng/ml, 12 hr). (F) Axonal length was measured after transfection of neurons with JIP1 siRNA (50 nM) for 48 hr and then incubation with netrin-1 (150 ng/ml) for 12 hr. All data are expressed as percent of respective controls (mean ± SEM, from six to eight independent experiments and ∼100 axons measured for each condition). Statistical significance was determined via one-way ANOVA, with post hoc Tukey's test for comparison between groups. Values of p < 0.05 (^∗^) were taken to be statistically significant, (^∗∗^) indicates values of p < 0.001.

Having determined the c-Abl phosphorylation site on JIP1, it was important to address how this phosphorylation event affected axonal development. The nonphosphorylatable JIP1-Y278F mutant failed to promote axonal growth (Figure 4A), whereas a JIP1-Y278D mutant, which can potentially mimic the phosphorylated state, increased axonal length to levels comparable to wild-type JIP1 (Figure 4A). In further studies, we found that wild-type JIP1 and the JIP1-Y278F mutant failed to increase axonal length in the presence of the c-Abl inhibitor STI571, whereas the JIP1-Y278D-mediated increase in axonal length was not affected by either the inhibitor or c-Abl siRNA (Figures 4B and 4C). Taken together, our results provide the first evidence for the direct tyrosine phosphorylation of JIP1 by c-Abl and we identify Tyr278 as the major site required for the regulation of JIP1-dependent axonal growth.

The results obtained with the JIP1 mutants were not due to mislocalization of the protein, given that the JIP1-Y278F mutant was still capable of binding to kinesin in vitro (data not shown) and we did not observe any significant changes in its localization compared to wild-type JIP1 (Figure S4D). Moreover, neither the inhibition nor the knockdown of c-Abl affected JIP1 localization to the axonal growth cones (data not shown). These observations suggest that the c-Abl regulation of JIP1 function is most likely to occur at the tips of axonal growth cones where subtle changes in extracellular cues regulate the cytoskeleton and control axonal growth.

To assess the relevance of the JIP1 and c-Abl interaction in a known model of axonal growth, we used netrin-1 stimulation to promote axonal elongation in culture. In vitro netrin-1 has been shown to increase the growth of thalamocortical axons [25] and to promote axonal branching [26], whereas in *Drosophila* Abl has been implicated as an effector of the netrin receptor Fra [27]. Here, we demonstrate that netrin-1 increases axonal length and that this was blocked by both pharmacological inhibition of c-Abl activity and c-Abl siRNA (Figure 4D), as well as by siRNA directed against JIP1 (Figure 4F). Crucially, the stimulation of cortical neurons with netrin-1 led to an increase in the tyrosine phosphorylation of endogenous JIP1 which was abrogated by c-Abl siRNA (Figure 4E).

Taken together, our results demonstrate novel roles for JIP1 as a specific regulator of axonal development and as a downstream target of a netrin and c-Abl mediated signaling pathway leading to axonal growth. The kinesin-1-dependent localization of JIP1 to developing axonal growth cones and its colocalization with dynamic microtubules and exploratory processes emanating from the growth cone suggest that it may be important for the regulation of cytoskeletal structures. c-Abl is a well-established regulator of cytoskeletal dynamics, and JIP1 may be an important downstream effector of c-Abl in cytoskeletal reorganization. The JIP1 scaffold protein, therefore, has the potential to act as a crucial link between extracellular signals and the regulation of cytoskeletal dynamics that lead to axonal development.

## Supplemental Data

Document 1. Experimental Procedures and six figures
