# Supplementary material for: The JIP1 Scaffold Protein Regulates Axonal Development in Cortical Neurons
Source: Curr Biol. 2008 Feb 12;18(3):221–6. doi: 10.1016/j.cub.2008.01.025 (PMC2258314; doi:10.1016/j.cub.2008.01.025)
Supplement: Document 1. Experimental Procedures and six figures [file mmc1.pdf]

Supplemental Data

The JIP1 Scaffold Protein Regulates Axonal Development in Cortical Neurons

Federico Dajas-Bailador, Emma V. Jones, and Alan J. Whitmarsh

Supplemental Experimental Procedures

Cell Culture and Transfections

Primary cortical neurons were prepared as described previously [S1] with minor modifications. In brief, mouse cortices were dissected from C57/BL6 embryonic day 17 mice and carefully triturated, and the cell suspension was plated onto poly-L-ornithine-coated culture plates. Neurons were cultured in Neurobasal medium (Invitrogen, supplemented with 5mM glutamine and 2% B-27 supplement) for a maximum of 15 days. Cells were treated with c-Abl inhibitor STI571 (Novartis), GSK3 inhibitor SB415286 (Sigma), taxol (Sigma), cytochalasin D (Sigma), and netrin-1 (R&D Systems). Neuronal transfections were performed via the Nucleofector electroporation system (Amaxa Biosystems), for siRNA transfections; or Lipofectamine 2000 (Invitrogen), for DNA plus or minus siRNA transfections. Where indicated, neurons were transfected with siRNA immediately after being plated on noncoated dishes and transferred to poly-L-ornithine-coated culture dishes 24 hr later. JIP1 siRNAs were from Santa Cruz Biotechnology (sc-35723, defined as A in figures) and Dharmacon (MQ-042191-00, defined as B in figures). Unless otherwise indicated, JIP1 siRNA-A was used in the experiments. c-Abl siRNA was from QIAGEN (SI00887131), and Arg siRNA was from Santa Cruz Biotechnology (sc-38946). Nonspecific control siRNA was from MWG: 5′-AGGUAGUGUAAUCGCCUUGTT-3′. COS-7 cells were maintained in Dulbecco's modified Eagle's medium supplemented with 5 mM glutamine, 100 units/ml penicillin/streptomycin, and 5% fetal bovine serum (Invitrogen). NIE-115 neuroblastoma cells were cultured similarly except that 10% fetal bovine serum was used. Cell lines were transfected via Lipofectamine (Invitrogen).

Expression Constructs

The expression vectors pcDNA3-T7-JIP1 and pcDNA3-T7-JIP2 were described previously [S2, S3]. pEBB-c-Abl ΔSH3 (c-Abl-CA), pcDNA3-Flag-Arg, and pcDNA3-T7-JIP3 were generous gifts from B. Mayer (University of Connecticut), D. Kufe (Harvard Medical School), and R. Davis (UMASS Medical Center), respectively. pMAX-eGFP was from Amaxa Biosystems. Site-directed mutagenesis was performed with the QuikChange kit (Stratagene), and mutations were verified by DNA sequencing. The expression vector pCDNA3-myc-hJIP1 (human) was generated by PCR of the human JIP1 cDNA from IMAGE clone 5260971 and subcloned with an N-terminal 6× myc tag into pCDNA3. The JIP1 mutants prepared in this study were pcDNA3-T7-JIP1-Y278F, pcDNA3-T7-JIP1-Y278D, pcDNA3-myc-hJIP1-R160G/P161G (based on [S4]), and pcDNA3-T7-JIP1-Y705A (based on [S5]).

Immunofluorescence and Image Analysis

Cortical neurons were fixed with 4% paraformaldheyde, and the following antibodies were used: anti-JIP1 (1:100, B7, Santa Cruz, sc-25267), anti-JIP1 (1:100) [S3], anti-MAP (1:1000, Sigma), and anti-Tau-1 (1:100, Chemicon). Secondary antibodies were Alexa 488 or 594-labeled goat anti-mouse (isotype specific) or anti-rabbit (Invitrogen). Images were obtained with an Olympus BX51 microscope attached to a CCD Coolsnap-ES camera (Photometrics, United Kingdom). For the measurement of axons, neurons were fixed at 3–4 days in vitro (d.i.v.), and images from random fields for each condition were taken from at least six independent neuronal preparations. An axon was defined as a neurite that was longer than 80 μm and at least three times the length of other neurites (in parallel, axons were also identified as neurites with JIP1-positive tips). Axon length was measured with Image J (http://rsb.info.nih.gov/ij/), and data are expressed as percent of respective controls (~100 axons measured for each condition from six to eight independent experiments). The average length of axons in control groups was 282 μm ± 22 (mean ± standard error of the mean [SEM]). Dendrite length was also measured in control and JIP1 siRNA experiments, and data were expressed as the mean ± SEM from five to seven independent experiments and ~300 dendrites for each condition. Average dendrite length in control groups was 48 μm ± 0.85. Unlike the effect observed on axons, the length of dendrites was not modified by the JIP1 siRNA (47.7 μm ± 0.82). Statistical significance was determined via one-way ANOVA, with post hoc Tukey's test for comparison between groups. Values of $p < 0.05$ (*) were taken to be statistically significant, (**) indicates values of $p < 0.001$.

Immunoprecipitations and GST Pull-Downs

Cells were washed once with ice-cold phosphate-buffered saline and then lysed in buffer containing 20 mM Tris, pH 7.4, 137 mM NaCl, 25 mM

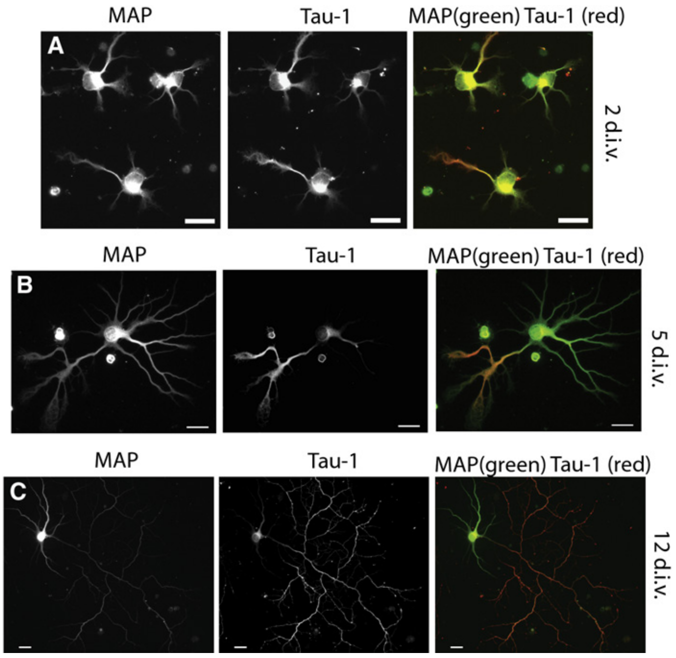

Figure S1. Localization of the Axonal Marker Tau-1 in Developing Axons

Cortical neurons were immunostained with MAP (green) and Tau-1 (red) antibodies at different stages of neuronal development.

(A) At 2 d.i.v., Tau-1 staining was present in multiple neurites and the soma.

(B) At 5 d.i.v., Tau-1 selectively localized to a single neurite that could be morphologically defined as the axon.

(C) At 12 d.i.v., Tau-1 selectively localized to the axon of mature cortical neurons. Scale bar represents 20 μm.

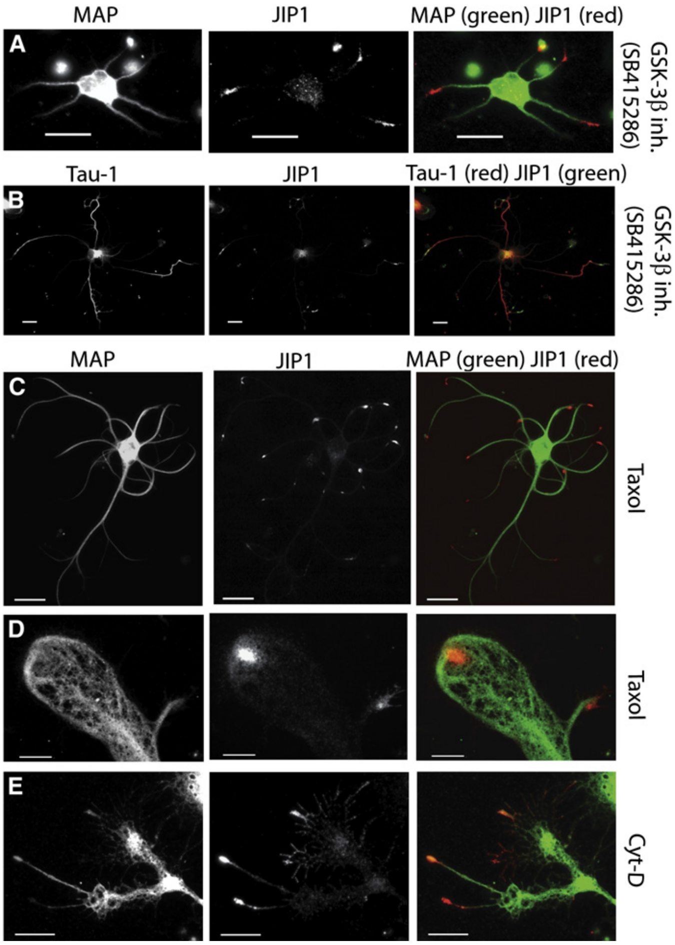

Figure S2. Relocalization of JIP1 after Induction of Supernumerary Axons and the Disruption of Cytoskeleton Dynamics

The formation of axon-dendrite polarity can be affected by a variety of experimental manipulations [S6–S10]. In particular, it has recently been demonstrated that inhibition of glycogen synthase-3β activity resulted in the formation of multiple axons [S11].

(A) At 2 d.i.v., the inhibition of GSK-3 activity (1 μM SB415286, 24 hr) in cortical neurons induced JIP1 localization to multiple neurites.

(B) At 5 d.i.v.. staining with the axonal marker Tau-1 (red) confirmed the generation of supernumerary axons after GSK-3 inhibition. Under these conditions, JIP-1 (green) localized to all of the Tau-1-positive neurites. Scale bars represent 20 μm.

(C) Neuronal polarization has also been shown to depend on microtubule-dynamics changes, which are necessary to maintain the differences between the initial axonal segment and the dendrites and to allow the development of polarized axonal transport [S9]. Inhibition of microtubule dynamics with taxol (1 μM, 24 hr) promoted JIP1 (red) localization to multiple neurites. Scale bar represents 20 μm.

(D) Higher-magnification analysis of axons showing JIP1 localization to central areas of axonal growth cones after short-term disruption of microtubule dynamics with taxol (10 μM, 2 hr). Scale bars represent 10 μm.

(E) The depletion of actin filaments with cytochalasin D (Cyt-D, 1 μM, 2 hr) promotes a drastic change in axonal growth-cone structure, with a significant increase in JIP1-enriched microtubule protrusions. Scale bars represent 10 μm. Together, these results confirm the specific localization of JIP1 to axons and its preferential association with dynamic microtubules capable of protruding into peripheral growth-cone areas.

β-glycero-phosphate, 2 mM sodium pyrophosphate, 2 mM EDTA, 1% Triton X-100, 10% glycerol, 1 mM phenylmethylsulfonyl fluoride, 1 mM sodium orthovanadate, and 5 μg/ml leupeptin. Lysates were centrifuged at 14,000 × g for 10 min to remove insoluble material. Epitope-tagged proteins were immunoprecipitated from lysates by incubation with anti-T7 tag antibody (Novagen) and protein A-Sepharose beads (Sigma) for 3 hr at 4°C. GST proteins were isolated by incubation of lysates with glutathione-Sepharose 4B (Amersham Biosciences). Beads were washed three times in lysis buffer,

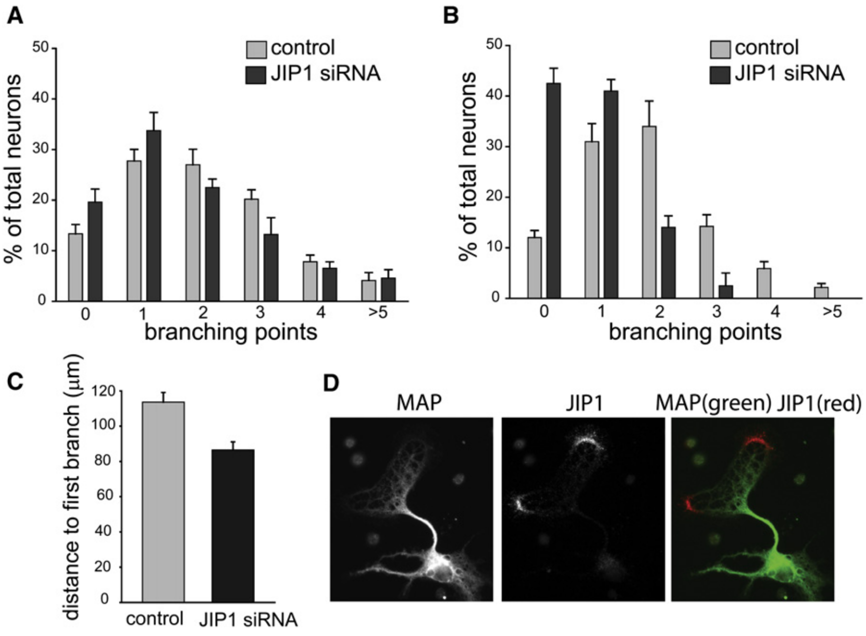

Figure S3. JIP1 Knockdown Affects Axonal Branch Formation

Axonal growth is required for development of correct neuron connectivity, a process which also involves the extension of branching processes either by bifurcation or interstitial development. It has been previously suggested that the extension of growth cones and axonal branches may involve similar signaling cues and cytoskeletal reorganization [S12, S13].

(A) Cortical neurons were transfected with JIP1 siRNA 24 hr after being plated, and 72 hr later the neurons were evaluated according to an index of 0 to >5 branching points. In this protocol, a small increase in the number of neurons with no axonal branches was observed.

(B and C) In developmentally delayed cortical neurons (see Supplemental Experimental Procedures) where a significant knockdown of JIP1 protein levels by siRNA has occurred prior to axonal differentiation, there was a substantial increase in the percent of neurons with no axonal branches and a decrease in the distance from the cell body to the first branching point.

(D) Immunolabelling of a cortical neuron at 5 d.i.v. showing JIP1 (red) at the tips of an axon during branching.

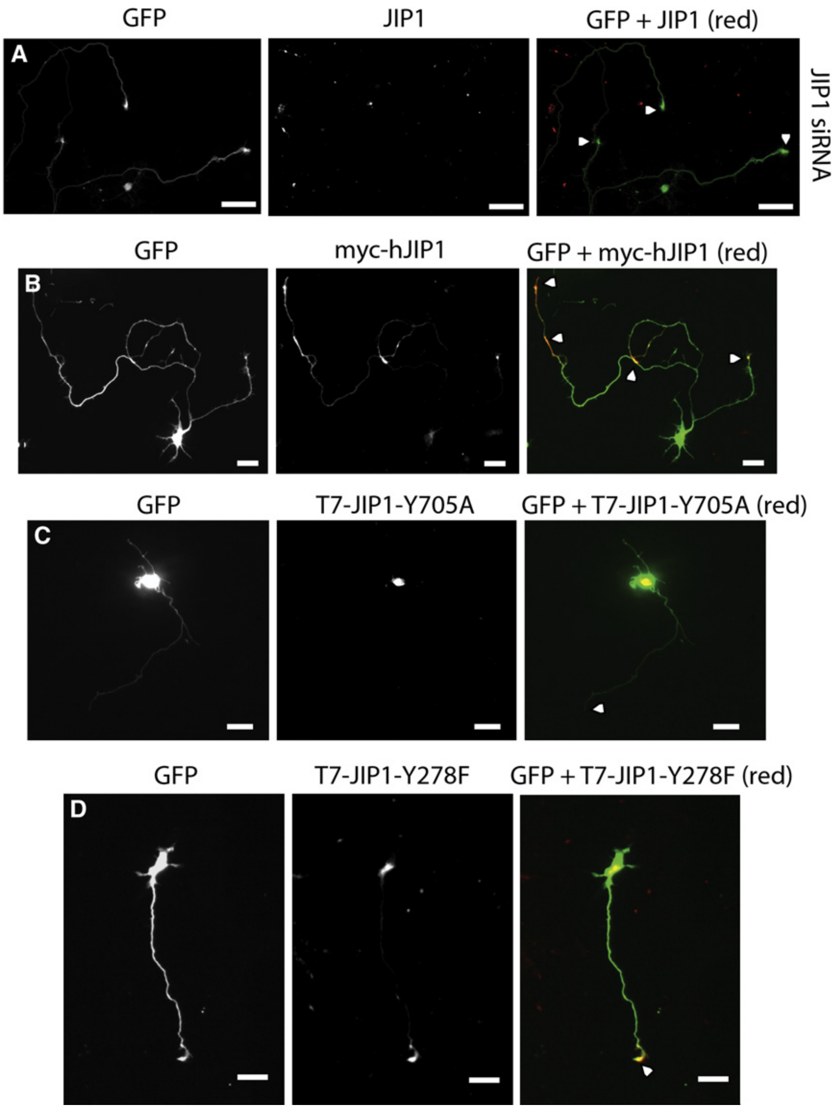

Figure S4. Localization of Endogenous JIP1 and Ectopically-Expressed JIP1 in Cortical Neurons at 3–4 D.I.V.

(A) Knockdown of JIP1 protein levels by siRNA in GFP-positive axonal growth cones detected with anti-JIP1 (B7; Santa Cruz Biotechnology). A similar result was obtained with a different monoclonal anti-JIP1 [S3] (data not shown).

(B) Ectopically-expressed JIP1 predominantly localizes to the tips of axonal processes with some staining of the cell body.

(C) The T7-tagged JIP1-Y705A mutant, which does not bind to kinesin-1, fails to localize to the tips of axonal processes.

(D) The T7-tagged JIP1-Y278F mutant lacking the c-Abl phosphorylation site still localizes to axonal growth cones. Arrows highlight the growth cones. Scale bar represents 20 μm.

and bound proteins were eluted by the addition of 6× SDS loading buffer. Endogenous JIP1 was immunoprecipitated with anti-JIP1 (B7, Santa Cruz Biotechnology) and protein G-sepharose beads (Amersham Biosciences).

Immunoblotting

Samples were resolved by SDS-PAGE (10% gels) and transferred to Immobilon-P membranes (Millipore), which were immunoblotted with the following antibodies: anti-T7 tag (Novagen), anti-myc (Santa Cruz Biotechnology), anti-GST (Amersham Biosciences), anti-β-actin (Abcam, United Kingdom), anti-phosphotyrosine (Transduction Labs), anti-c-Abl (BD Biosciences), anti-JIP1 (B7), anti-Arg, and anti-phosphotyrosine (Santa Cruz Biotechnology). Primary antibodies were detected with HRP-conjugated secondary antibodies (Amersham Biosciences) followed by enhanced chemiluminescence (Pierce).

In Vitro Protein Kinase Assay

Assays were performed with recombinant c-Abl (Upstate) and 2 μg of the GST-fusion protein GST-JIP1-JBD(127-281), GST-JIP1-JBD(127-281) Y278F, or GST-CRKI(102-304) expressed in and purified from *E.coli*. The proteins were incubated in kinase assay buffer (40 mM MOPS [pH 7.2], 1mM EDTA, 30 mM MgCl2, and 0.5 mM DTT) together with 50 μM [γ32ATP] (10Ci/nmol) for 30 min. The reactions were terminated with the addition of Laemmli buffer and the phosphorylation of substrates examined by SDS-PAGE and autoradiography.

Supplemental References

S1. Hejmadi, M.V., Dajas-Bailador, F., Barns, S.M., Jones, B., and Wonnacott, S. (2003). Neuroprotection by nicotine against hypoxia-induced apoptosis in cortical cultures involves activation of multiple nicotinic acetylcholine receptor subtypes. Mol. Cell. Neurosci. *24*, 779–786.

S2. Whitmarsh, A.J., Cavanagh, J., Tournier, C., Yasuda, J., and Davis, R.J. (1998). A mammalian scaffold complex that selectively mediates MAP kinase activation. Science *281*, 1671–1674.

S3. Yasuda, J., Whitmarsh, A.J., Cavanagh, J., Sharma, M., and Davis, R.J. (1999). The JIP group of mitogen-activated protein kinase scaffold proteins. Mol. Cell. Biol. *19*, 7245–7254.

S4. Nihalani, D., Wong, H.N., and Holzman, L.B. (2003). Recruitment of JNK to JIP1 and JNK-dependent JIP1 phosphorylation regulates JNK module dynamics and activation. J. Biol. Chem. *278*, 28694–28702.

S5. Verhey, K.J., Meyer, D., Deehan, R., Blenis, J., Schnapp, B.J., Rapoport, T.A., and Margolis, B. (2001). Cargo of kinesin identified as JIP scaffolding proteins and associated signaling molecules. J. Cell Biol. *152*, 959–970.

S6. Dotti, C.G., and Banker, G.A. (1987). Experimentally induced alteration in the polarity of developing neurons. Nature *330*, 254–256.

S7. Schwamborn, J.C., and Puschel, A.W. (2004). The sequential activity of the GTPases Rap1B and Cdc42 determines neuronal polarity. Nat. Neurosci. *7*, 923–929.

S8. Bradke, F., and Dotti, C.G. (1999). The role of local actin instability in axon formation. Science *283*, 1931–1934.

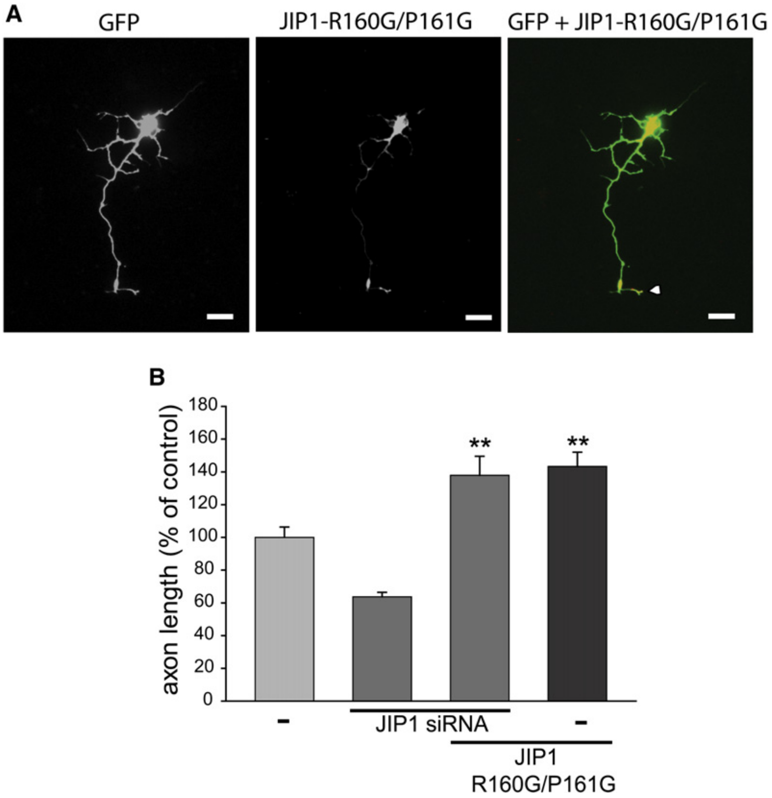

Figure S5. Localization and Axonal Growth Properties of the JIP1-R160G/P161G Mutant Lacking the JNK-Binding Site

(A) The JIP1-R160G/P161G mutant predominantly localizes to the tip of the axonal growth cone in 4 d.i.v. cortical neurons (see arrow) with some staining of the cell body. Scale bar represents 20 μm.

(B) Similar to wild-type JIP1, the ectopic expression of the human JIP1-R160G/P161G mutant increased axonal growth and rescued the decreased axonal growth caused by the JIP1 siRNA. Data are expressed as percent of respective controls (mean ± standard error of the mean [SEM], from five independent experiments and ~100 axons measured for each condition). Statistical significance was determined via one-way ANOVA, with post hoc Tukey's test for comparison between groups. Values of $p < 0.05$ (*) were taken to be statistically significant, (**) indicates values of $p < 0.001$.

S9. Nakata, T., and Hirokawa, N. (2003). Microtubules provide directional cues for polarized axonal transport through interaction with kinesin motor head. J. Cell Biol. *162*, 1045–1055.

S10. Jacobs, T., Causeret, F., Nishimura, Y.V., Terao, M., Norman, A., Hoshino, M., and Nikolic, M. (2007). Localized activation of p21-activated kinase controls neuronal polarity and morphology. J. Neurosci. *27*, 8604–8615.

S11. Jiang, H., Guo, W., Liang, X., and Rao, Y. (2005). Both the establishment and the maintenance of neuronal polarity require active mechanisms: critical roles of GSK-3β and its upstream regulators. Cell *120*, 123–135.

S12. Kalil, K., Szebenyi, G., and Dent, E.W. (2000). Common mechanisms underlying growth cone guidance and axon branching. J. Neurobiol. *44*, 145–158.

S13. Dent, E.W., Barnes, A.M., Tang, F., and Kalil, K. (2004). Netrin-1 and semaphorin 3A promote or inhibit cortical axon branching, respectively, by reorganization of the cytoskeleton. J. Neurosci. *24*, 3002–3012.

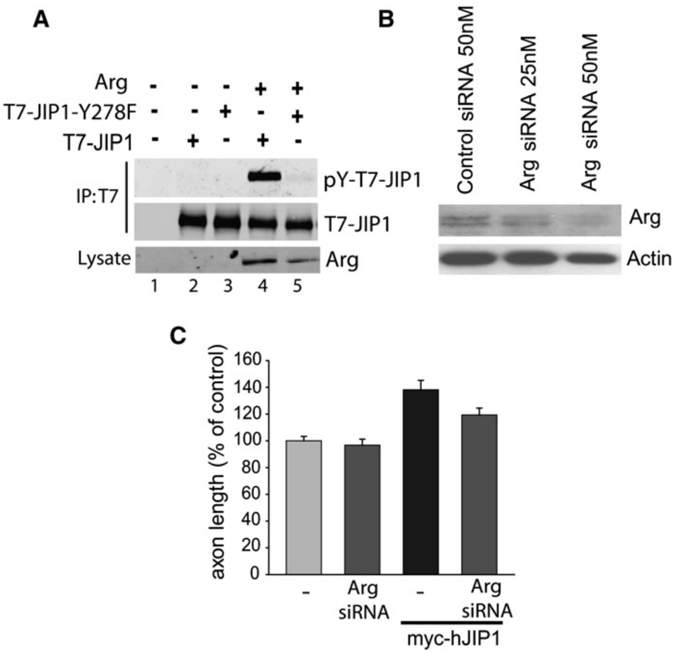

Figure S6. The Contribution of Arg to JIP1-Mediated Axonal Growth

(A) Arg kinase phosphorylates JIP1 at Tyr278 in vitro. Constructs expressing T7-JIP1 and T7-JIP1-Y278F were introduced into COS-7 cells with or without an Arg-expressing vector. JIP1-containing complexes were immunoprecipitated with anti-T7 tag antibody, and the presence of Tyr-phosphorylated JIP1 was analyzed with phosphotyrosine-specific antibodies.

(B) Immunoblot for Arg protein levels after transfection with Arg siRNA.

(C) Measurement of axon length in neurons transfected with Arg siRNA plus or minus a construct expressing myc-tagged human JIP1. The knockdown of Arg protein levels did not affect basal axonal growth, but produced a small decrease in the JIP1-dependent increase in axon length. Data are expressed as percent of respective controls (mean ± SEM, from five independent experiments and ~100 axons measured for each condition). Statistical significance was determined via one-way ANOVA, with post hoc Tukey's test for comparison between groups. Values of $p < 0.05$ (*) were taken to be statistically significant, (**) indicates values of $p < 0.001$.
